# Fermented Oyster Extract Promotes Osteoblast Differentiation by Activating the Wnt/β-Catenin Signaling Pathway, Leading to Bone Formation

**DOI:** 10.3390/biom9110711

**Published:** 2019-11-06

**Authors:** Ilandarage Menu Neelaka Molagoda, Wisurumuni Arachchilage Hasitha Maduranga Karunarathne, Yung Hyun Choi, Eui Kyun Park, You-Jin Jeon, Bae-Jin Lee, Chang-Hee Kang, Gi-Young Kim

**Affiliations:** 1 Department of Marine Life Science, Jeju National University, Jeju 63243, Korea; neelakagm2012@gmail.com (I.M.N.M.); hasikarunarathne@gmail.com (W.A.H.M.K.); youjinj@jejunu.ac.kr (Y.-J.J.); 2 Department of Biochemistry, College of Oriental Medicine, Dong-Eui University, Busan 47227, Korea; choiyh@deu.ac.kr; 3 Department of Oral Pathology and Regenerative Medicine, School of Dentistry, Institute for Hard Tissue and Biotooth Regeneration, Kyungpook National University, Daegu 41940, Korea; epark@knu.ac.kr; 4 Marine Bioprocess Co., Ltd., Busan 46048, Korea; hansola82@hanmail.net; 5 Bioresources Industrialization Support Department, Nakdonggang National Institute of Biological Resources, Sangju 37242, Korea; ckdgml3735@nnibr.re.kr

**Keywords:** *Crassostrea gigas*, oyster, bone formation, mineralization, Wnt/β-catenin

## Abstract

The Pacific oyster, *Crassostrea gigas*, is well-known as a nutritious food. Recently, we revealed that fermented extract of *C. gigas* (FO) inhibited ovariectomy-induced osteoporosis, resulting from suppression of osteoclastogenesis. However, since the beneficial effect of FO on osteogenesis is poorly understood, it was examined in mouse preosteoblast MC3T3-E1 cells, human osteosarcoma MG-63 osteoblast-like cells, and zebrafish larvae in this study. We found that FO increased mitochondrial activity from days 1 to 7; however, total cell number of MC3T3-E1 cells gradually decreased without any change in cell viability, which suggests that FO stimulates the differentiation of MC3T3-E1 cells. FO also promoted the expression of osteoblast marker genes, including *runt-related transcription factor 2 (mRUNX2)*, *alkaline phosphatase (mALP)*, *collagen type I α1 (mCol1α1)*, *osteocalcin (mOCN)*, *osterix (mOSX)*, *bone morphogenetic protein 2 (mBMP2)*, and *mBMP4* in MC3T3-E1 cells accompanied by a significant increase in ALP activity. FO also increased nuclear translocation of RUNX2 and OSX transcription factors, ALP activity, and calcification in vitro along with the upregulated expression of osteoblast-specific marker proteins such as RUNX2, ALP, Col1α1, OCN, OSX, and BMP4. Additionally, FO enhanced bone mineralization (calcein intensity) in zebrafish larvae at 9 days post-fertilization comparable to that in the β-glycerophosphate (GP)-treated group. All the tested osteoblast marker genes, including *zRUNX2a*, *zRUNX2b*, *zALP*, *zCol1a1*, *zOCN*, *zBMP2*, and *zBMP4*, were also remarkably upregulated in the zebrafish larvae in response to FO. It also promoted tail fin regeneration in adult zebrafish as same as the GP-treated groups. Furthermore, not only FO positively regulate β-catenin expression and Wnt/β-catenin luciferase activity, but pretreatment with a Wnt/β-catenin inhibitor (FH535) also significantly decreased FO-mediated bone mineralization in zebrafish larvae, which indicates that FO-induced osteogenesis depends on the Wnt/β-catenin pathway. Altogether, the current study suggests that the supplemental intake of FO has a beneficial effect on osteogenesis.

## 1. Introduction

Bones are metabolically-active mineralized connective tissue that undergo continuous remodeling throughout their life via osteoclast-mediated bone resorption and osteoblast-mediated bone formation [1,2]. However, imbalance in bone remodeling increases bone resorption and decreases bone formation [3], which causes osteoporosis characterized by bone mass reduction and microarchitectural deterioration, bone fractures, morbidity, and eventually shortened lifespans [4,5]. Effective prevention and treatment of osteoporosis and bone fractures are paramount, since patients with osteoporosis may be completely asymptomatic until they experience a fracture [6]. Therefore, many scientists have tried to find effective compounds and molecules that stimulate osteoblast differentiation and inactivate osteoclast vitalization [7,8].

Osteoblasts originate from mesenchymal stem cells that are located in the marrow, endosteum, periosteum, and bone canals [9], whereas osteoclasts originate from hematopoietic stem cells and are generally located on the bone surface [10]. Although osteoblasts are present throughout an individual’s life, their activity is highest during embryonic skeletal development [9]. Notably, a dynamic balance between bone formation and bone resorption, which is controlled by calciotropic hormones and cytokines, is required for skeletal integrity [11]. Osteoblast activation in an adult organism is associated with bone regeneration defects and depletion of the bone matrix [12]. Osteoblast differentiation is also associated with a number of factors such as bone morphogenetic proteins (BMPs), transforming growth factor-β (TGF-β), glucocorticoids, insulin-like growth factor I (IGF-I), and vascular endothelial growth factor (VEGF) [13,14,15]. Moreover, several molecules are essential for the development and maintenance of mineralized skeletal elements. Matrix synthesis in osteoblast culture models is shown to activate osteoblastic markers, such as runt-related transcription factor2 (RUNX2), alkaline phosphatase (ALP), collagen type I α1 (Col1α1), osteocalcin (OCN), osterix (OSX), and Wnt/β-catenin [16,17].

Oysters are among the popular type of edible shellfish in the world because of their nutritional content. Recently, several studies demonstrated that oyster shell components enhanced bone tissue regeneration due to their high calcium content [18,19]. Chen et al. [20] confirmed that peptides of *Crassostrea gigas* promoted the activity of ALP, which is a specific osteoblast differentiation marker in preosteoblast cells, by binding to integrin α5β1; however, they neither investigated the occurrence of the peptides-mediated osteogenesis in vivo, nor did they determined the signaling pathways related to osteogenesis. Furthermore, enzyme-digested proteins in shellfish are known to help the absorption of functional nutrients in the intestines of mammals and increase their functionality in the body [21]. Recently, we determined that fermented oyster (*C. gigas*) extract (FO) exerted protective effects against receptor activator of nuclear factor-κB ligand (RANKL)-induced osteoclastogenesis [22]. Additionally, our research team found that FO prevented ovariectomy-induced bone loss accompanied by the inhibition of osteoclast [23]. Therefore, determining the osteogenic effect of FO will help to understand whether *C. gigas* is a good nutritional and therapeutic food source in terms of promoting bone formation.

In the current study, we confirmed that FO activated the expression of osteogenesis-related genes such as *RUNX2*, *ALP*, *Col1α1*, *OCN*, *OSX*, and *BMPs*, in preosteoblast MC3T3-E1 cells, MG-63 cells, and zebrafish larvae. Furthermore FO increased nuclear translocation of RUNX2 and OSX transcription factors, ALP activity, and in vitro calcification along with the upregulated expression of osteoblast-specific marker proteins such as RUNX2, ALP, Col1α1, OCN, OSX, and BMP4 in MG-63 cells. FO treatment also enhanced bone mineralization (calcein intensity) in zebrafish larvae at 9 days post-fertilization (dpf) and promoted tail regeneration in adult zebrafish at 6 and 12 days post-amputation (dpa). Furthermore, FO increased β-catenin expression and Wnt/β-catenin activity from days 3 to 7 due to the translocation of β-catenin to the nucleus. Moreover, the presence of the Wnt/β-catenin inhibitor, FH535, suppressed FO-mediated bone mineralization in zebrafish larvae. These results indicate that FO is potently functional in stimulating osteoblast activation and differentiation, which leads to osteogenesis.

## 2. Materials and Methods

### 2.1. Reagents and Antibody

3-(4,5-Dimethylthiazol-2-y-2,5-diphenyltetrazolium bromide (MTT), calcein, alizarin red, dexamethasone (DEX), gelatin, donkey serum, 4′6-diamidino-2-phenylindole (DAPI), and β-glycerophosphate (GP) were purchased from Sigma-Aldrich Chemical Co. (St. Louis, MO, USA). Specific antibodies against RUNX2, ALP, Col1α1, OCN, OSX, BMP4, β-actin, β-catenin, and nucleolin were purchased from Santa Cruz Biotechnology (Santa Cruz, CA, USA). Alexa Fluor^®^ 647 and Alexa Fluor^®^ 488 was purchased from Abcam (Cambridge, MA, UK). Dako faramount aqueous mounting media was purchased from Dako (Carpinteria, CA, USA). All other chemicals were supplied from Sigma-Aldrich Chemical Co. Minimum Essential Medium Alpha Modification (α-MEM), fetal bovine serum (FBS), and antibiotics mixtures were obtained from WelGENE (Gyeongsan-si, Gyeongsangbuk-do, Korea). Commercial FO (product name: FO100) was kindly supplied from Marine Bioprocess Co. (Busan-si, Korea). In brief, deshelled and frosted Pacific oyster (*C. gigas*) was obtained from Deokyeon Seafood Co. Ltd. (Tongyeong-si, Gyeongsangnam-do, Korea). Alcalase 2.4L FG (Novozyme, Bagsvaerd, Denmark) as a commercial protease was used to hydrolyze the oyster. γ-Gminobutyric acid (GABA)-producing *Lactobacillus brevis* BJ20 (Accession No. KCTC 11377BP) was inoculated into the seed media (yeast extract 3%, glucose 1%, monosodium glutamate 1%, and water 95%) and then fermented.

### 2.2. Proximate Analysis of FO

Protein was measured using automatic Kjeltec Analyzer Unit 2300 (Foss Teor, Hoganas, Sweden) and lipid was calculated according to the standard procedure [24]. Ash content was determined after combustion at 550 °C for 4 h in a muffle furnace. Carbohydrate was measured by a phenol-sulfuric acid method [25]. Amino acid content of FO was determined using Dionex UltiMate 3000 HPLC and UHPLC systems (Thermo Fisher Scientific, Waltham, MA, USA) at the National Instrumentation Center for Environmental Management (Seoul National University, Seoul, Korea).

### 2.3. Cell Culture and MTT Activity

Mouse preosteoblast MC3T3-E1 cells and osteosarcoma MG-63 human osteoblast-like cells were obtained by the American Type Culture Collection (ATCC, Manassas, VA, USA) and maintained in α-MEM supplemented with 10% FBS and antibiotics mixture in a humidified incubator at 5% CO_2_ and 37 °C. In order to assess the MTT activity, MC3T3-E1 cells were seeded into 24-well plates at a density of 3 × 10^3^ cells/mL and incubated with different concentrations (0–200 μg/mL) of FO for 1, 3, 5, and 7 days. DEX (100 nM) was used for a positive control for MC3T3-E1 differentiation. Relative MTT activity was measured by incubating 0.5 mg/mL MTT. Formazan was dissolved with DMSO and the absorption at 540 nm was determined by an enzyme-linked immunosorbent assay (ELISA) microplate reader (Thermo Fisher Scientific).

### 2.4. Flow Cytometry Analysis

Cell viability and total cell numbers were measured by flow cytometric analysis. Briefly, MC3T3-E1 cells were seeded at 3 × 10^3^ cells/mL in six well plates for overnight and treated with the different concentrations (0–200 μg/mL) of FO for 1, 3, 5, and 7 days. DEX (100 nM) was used a positive control for MC3T3-E1 cell viability. After harvesting, the cells were washed with ice-cold phosphate-buffered saline (PBS) and incubated with Muse^®^ cell count and viability kit (EMD Millipore, Billerica, MA, USA) for 5 min. Cell viability and total cell number were measured by Muse^®^ cell cycler (EMD Millipore).

### 2.5. Reverse Transcriptase-Polymerase Chain Reaction (RT-PCR)

MC3T3-E1 cells were treated with the different concentrations of FO for the indicated days and then total RNA was extracted using easy-BLUE™ total RNA extraction kit (iNtRON Biotechnology, Sungnam-si, Gyeonggi-do, Korea) according to the manufacturer’s instruction. Two micrograms of RNA were reverse-transcribed using MMLV reverse transcriptase (Bioneer; Daejeon, Korea). The target cDNA was amplified using the following mouse primers of *mRUNX2* (forward 5′-CAT GGT GGA GAT CAT CGC GG-3′ and reverse 5′-GGC CAT GAC GGT AAC CAC AG-3′), *mALP* (forward 5′-TTG TGG CCC TCT CCA AGA CA-3′ and reverse 5′-GAC TTC CCA GCA TCC TTG GC-3′), *mCol1α1* (forward 5′-GAC GCA TGG CCA AGA AGA CA-3′ and reverse 5′-TCT TTG GGG GTT GGG ACA GT-3′), *mOCN* (forward 5′-GCC CTG AGT CTG ACA AAG CC-3′ and reverse 5′-GCG TTT GTA GGC GGT CTT CA-3′), *mOSX* (forward 5′-AAG GCG GTT GGC AAT AGT GG-3′ and reverse 5′-GCA GCT GTG AAT GGG CTT CT-3′), *mBMP2* (forward 5′- TGC TGA CCA CCT GAA CTC CA-3′ and reverse 5′-CAG CCC TCC ACA ACC ATG TC-3′), *mBMP4* (forward 5′-GAC TTC ACT GAC GTG GGC TG-3′ and reverse 5′-TGG GGA CAC AAC AGG CCT TA-3′), and *mGAPDH* (forward 5′-CGA TGC CCC CAT GTT TGT TGT GA-3′ and reverse 5′-ACA GTC TTC TGG GTG GCA GT-3′).

Zebrafish larvae at 3 days post-fertilization (dpf) were treated with the indicated concentrations of FO and mRNA was extracted using an easy-BLUE™ total RNA extraction kit at 9 dpf. The target cDNA was amplified using the following the zebrafish primers of *zRUNX2a* (forward 5′-GAC GGT GGT GAC GGT AAT GG-3′ and reverse 5′-TGC GGT GGG TTC GTG AAT A-3′), *zRUNX2b* (forward 5′-CGG CTC CTA CCA GTT CTC CA-3′ and reverse 5′-CCA TCT CCC TCC ACT CCT CC-3′), *zALP* (forward 5′-CAA GAA CTC AAC AAG AAC-3′ and reverse 5′-TGA GCA TTG GTG TTA TAC-3′), *zCol1a* (forward 5′-CTG TGC CAA TCC CAT CAT TTC-3′ and reverse 5′-ATA TCG CCT GGT TCT CCT TTC-3′), *zOCN* (forward 5′-TGG CCT CTA TCA TCA TGA GAC A-3′ and reverse 5′-CTC TCG AGC TGA AAT GGA GTC-3′), *zBMP2* (forward 5′-CGG CTC CTA CCA GTT CTC CA-3′ and reverse5′-CCA CTC CCC TCC ACT CCT CC-3′), *zBMP4* (forward 5′-TTG TGC TGT GCA TGT TTG AA-3′ and reverse 5′-GGT CGC TTG GCT ATG TGT TT-3′), and *zβ-actin* (forward 5′-CGA GCG TGG CTA CAG CTT CA-3′ and reverse 5′-GAC CGT CAG GCA GCT CAT AG-3′). The PCR products were separated by electrophoresis on 1.2% agarose gel and stained with 0.01% ethidium bromide visualized under UV light.

### 2.6. Alkaline Phosphatase (ALP) Activity

MC3T3-E1 cells and MG-63 cells were seeded in 24-well plates and then treated with the different concentrations of FO (50 µg/mL and 100 µg/mL) for 7 days. DEX (100 nM) was used as a positive control for osteoblast differentiation. ALP activity was measured by a tartrate-resistant acid phosphatase (TRACP) & alkaline phosphatase (ALP) double-staining Kit (Takara Bio Inc., Kusatsu, Shiga, Japan) according to the manufacture’s protocol. Briefly, the cells were rinsed three times with PBS and incubated with fixation buffer for 5 min. Then, ALP substrate was added into each well and incubated at 37 °C for 45 min. The images of each well were taken by Olympus camera OM-4T (Olympus Corp., Shinjuku, Tokyo, Japan). Density of the collected images was quantified by Image J software (National Institute of Health; Bethesda, MD, USA). Mineralization nodules were dissolved by cetylopyridinium chloride and the OD value was measured at 520 nm using an ELISA microplate reader (Thermo Fisher Scientific).

### 2.7. Alizarin Red Staining

In vitro calcium deposit assay, MG-63 cells were seeded in a 24 well plate at a density of 3 × 10^3^ cells/mL for overnight and then treated with different concentrations of FO (50 µg/mL and 100 µg/mL) for 3, 5, and 7 days. In vitro calcium deposit was measured by staining with 2% alizarin red. Briefly, MG-63 cells were washed with PBS and fixed with 4% paraformaldehyde for 30 min at 37 °C. Then, the cells were washed with PBS and stained with 2% alizarin red solution for 30 min. Images of each wells were taken with a phase contrast microscope (Ezscope i900PH, Macrotech; Goyang, Gyeonggi-do, Korea).

### 2.8. Protein Extraction and Western Blot Analysis

MC3T3-E1 cells and MG-63 cells were harvested and lysed with RIPA lysis buffer (iNtRON biotechnology). After cleaning lysates by centrifugation, protein was quantified by the Bio-Rad protein assay reagents (Bio-Rad, Hercules, CA, USA). In a parallel experiment, the cells were washed with ice-cold phosphate buffer saline (PBS), and cytosolic and nuclear proteins were extracted using NE-PER^TM^ Nuclear and Cytoplasmic Extraction Reagents (Pierce, Rockford, IL, USA). An equal amount of protein was separated by SDS-polyacrylamide gel, transferred onto nitrocellulose membrane (Schleicher & Schuell, Keene, NH, USA), and then immunoblotted with specific antibodies. The values were normalized with the intensity levels of β-actin and nucleolin for cytosolic and nuclear β-catenin.

### 2.9. RUNX2 and OSX Immunostaining

MG-63 cells (3 × 10^3^ cells/mL) were seeded on 3% gelatin-coated coverslips and allowed to attach on cover slips overnight. Then, the different concentrations of FO (50 µg/mL and 100 µg/mL) were treated for 7 days. DEX (100 nM) was used as the positive control. The cells were fixed with 4% paraformaldehyde for 10 min at 37 °C and washed three times with ice- PBS and permeabilized with 0.1% Triton X-100 for 10 min at room temperature followed by washing with ice-cold PBST (PBS + 0.1% tween 20) for 5 min each. The cells were blocked with 10% donkey serum and incubated with anti-RUNX2 and anti-OSX antibodies (1:100 in 10% donkey serum) overnight at 4 °C. After washing with ice-cold PBST, fluorescent dye-conjugated secondary antibody was added (Alexa Fluor^®^ 488 for anti-RUNX2 and Alexa Fluor^®^ 647 for anti-OSX), incubated for 2 h at room temperature, and washed three times with ice-cols PBST for 5 min each. Then, the cells were incubated with DAPI (300 nM) for 10 min and washed three times with ice-cold PBST for 5 min to remove excessive DAPI. The coverslips were mounted onto glass slides with Dako faramount aqueous mounting media and fluorescence images were captured by CELENA^®^ S digital imaging system (Logos biosystems, Anyang-si, Gyeonggi-do, Korea).

### 2.10. TOPFlash Luciferase Assay

MC3T3-E1 cells were seeded in 12 well plates and transfected with TOPFlash DNA (1 μg) using x-tremGENE9 (Promega, Madison, WI, USA). For co-transfection with Renilla luciferase, additional 0.1 μg of DNA was used. The luciferase activity was measured at 24 h using the Dual- Luciferase^®^ Reporter Assay System (Promega) according to the recommended protocol. The TOPFlash activity was normalized to Renilla luciferase signals and luciferase activity was measured via GloMax^®^ 96 Microplate Luminometer (Promega).

### 2.11. Bone Mineralization in Zebrafish Larvae

Zebrafish was raised and handled according to standard guidelines of the Animal Care and Use Committee of Jeju National University (Jeju-do, Korea). In order to monitor the vertebrae formation of zebrafish in vivo, a calcein green fluorescent marker was used. To visualize and analyze the newly formed vertebrae, zebrafish larvae at 3 dpf were treated with 50 µg/mL and 100 µg/mL FO for 6 days. GP (4 mM) was used as a positive control for zebrafish calcification. The media was changed every 2 days. At 9 dpf, the larvae were immersed in 0.05% calcein solution for 10 min and then rinsed in fresh water three times for 10 min in order to allow diffusion of the free calcein. After rinsing, the larvae were anesthetized in 0.04% tricaine methanesulfonate solution and mounted on depression slides using 2% methylcellulose before imaging.

### 2.12. Fin Regeneration in Adult Zebrafish

Four-month-old zebrafish was used for caudal fin amputations. Zebrafish were anaesthetized in 1% tricaine methanesulfonate and a small scissor was used for fin amputations, removing half of the fin. Various concentrations of FO (50 µg/mL and 100 µg/mL) and 4 mM GP were applied to 28 °C tank water. Then, 6 days after FO treatment, the zebrafish were stained with the 0.05% calcein for 15 min. At 12 days after FO treatment, the fish were stained with 0.02% alizarin red and the fluorescence images were collected. FO and E3 medium (34.8 g NaCl, 1.6 g KCl, 5.8 g CaCl_2_.2H_2_O, and 9.78 g MgCl_2_.6H_2_O in 1 L double-distilled water, pH 7.2 supplemented with 1% methylene) were changed every 2 days.

### 2.13. Statistical Analysis

The images were visualized with Chemi-Smart 2000 (Vilber Lourmat, Cedex, France). Images were captured using Chemi-Capt (Vilber Lourmat) and transported into Adobe Photoshop (version 8.0). All bands were quantified by Image J software. All data of RT-PCR and western blots were statistically analyzed by Sigma Plot 12.0 software. All data are presented as mean ± the standard error of the mean (SEM). Significant differences between groups were determined using the one-way analysis of variation (ANOVA) with Bonferroni correction. Values of *** *p* < 0.001, ** *p* < 0.01, and * *p* < 0.05 were considered to indicate statistical significance. The results shown in each of the figures in this article are representative of at least three independent experiments.

## 3. Results

### 3.1. FO Is Rich in Protein

The proximate analysis (Table S1) showed that FO is high in protein content (60.0 ± 0.7%) and carbohydrate (30.0 ± 0.7%) and contains a low content of lipid (3.3 ± 0.1%), while there was little ash content present (0.7 ± 0.1%). In the amino acid analysis, lysine and GABA were highly concentrated in FO (Table S2 and Figure S1).

### 3.2. FO Increases Mitochondrial Activity in Preosteoblast MC3T3-E1 Cells and Decreases Total Cell Number

**Figure 1:**
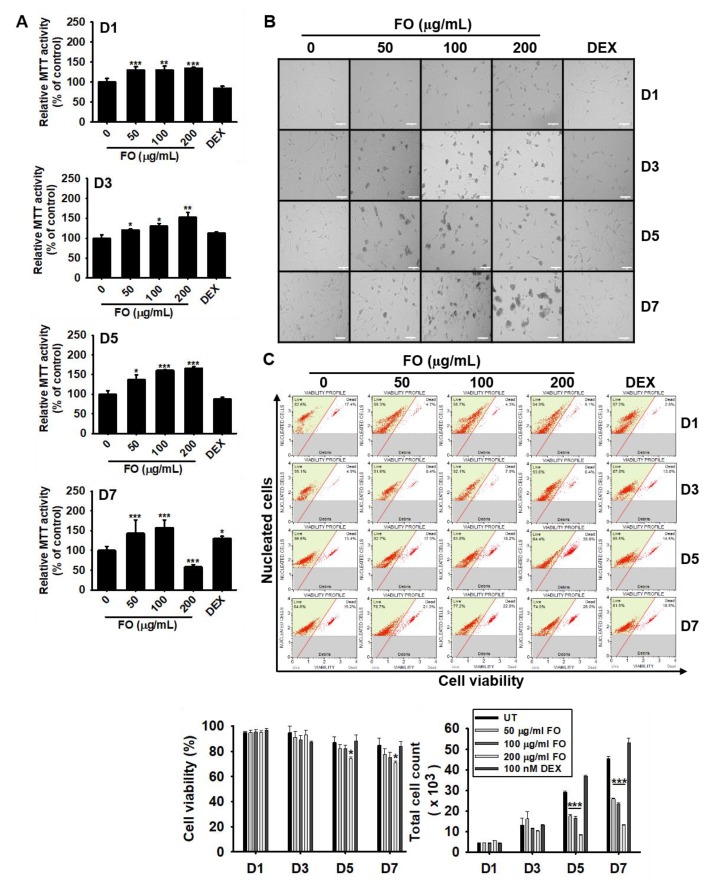
FO increases mitochondrial activity in preosteoblast MC3T3-E1 cells and decreases total cell number. MC3T3-E1 cells were treated with FO (0–200 µg/mL) or 100 nM DEX for 7 days. (**A**) Mitochondrial activity was measured on day 1 (D1), day 3 (D3), day 5 (D5), and day 7 (D7), and (**B**) cell images were collected by Ezscope i900PH phase contrast microscope (×10). Scale bar shows 100 μm. (**C**) Under the same experimental conditions, cell viability and total cell count were measured using flow cytometric analysis. Significant differences among the groups were determined using the one-way ANOVA followed by Bonferroni correction. All data are presented as mean ± SEM (*** *p* < 0.001, ** *p* < 0.01, and * *p* < 0.05 versus untreated group). FO; fermented extract of *C. gigas*. DEX; dexamethasone, and UT; untreated control.

To determine whether FO modulates mitochondrial activity, preosteoblast MC3T3-E1 cells were treated with various concentrations of FO for 7 days. Mitochondrial activity was measured using an MTT assay. FO, at concentrations below 100 µg/mL, gradually upregulated relative MTT activity up to day 7, while FO at 200 µg/mL increased MTT activity by day 5 and was significantly downregulated by 58.6 ± 5.4% at day 7 (Figure 1A). DEX (100 nM), used as a positive control, did not alter relative MTT activity up to day 5 and then moderately increased it at day 7 up to 130.4 ± 5.3%. Additionally, we observed that FO induced morphological changes in the cells, which appeared as clumps in the middle of the plate (Figure 1B). We also performed flow cytometric analysis to further confirm this phenomenon and found that FO slightly downregulated relative cell viability at day 5 [82.4 ± 2.8%, 72.0 ± 2.8%, and 74.1 ± 1.3% at 50 µg/mL, 100 µg/mL, and 200 µg/mL of FO when compared with that of untreated control (87.1 ± 4.4%)] and day 7 [77.3 ± 4.7%, 74.9 ± 4.5%, and 71.2 ± 1.5% at 50 µg/mL, 100 µg/mL, and 200 µg/mL of FO compared with the untreated control (84.9 ± 5.7%)] (Figure 1C, left-bottom). The total cell number significantly decreased in response to FO treatment when compared to the untreated group at days 5 and 7. The total cell count at day 5 was 29.3 ± 0.5 × 10^3^, 17.6 ± 0.8 × 10^3^, 16.7 ± 0.7 × 10^3^, and 8.4 ± 0.3 × 10^3^ cells at 0, 50 µg/mL, 100 µg/mL, and 200 µg/mL of FO, respectively (Figure 1C, right-bottom). Viability was observed to slightly decrease at day 7, and the total cell count significantly decreased accompanied by big cell clumps, which indicated that FO increased the induction of MC3T3-E1 cell differentiation. These results reveal that FO is not directly cytotoxic towards MC3T3-E1 cells, which differentiates into osteoblast cells, followed by cell clump formation and high mitochondrial activity.

### 3.3. FO Upregulates the Specific Marker Gene Expression Responsible for Osteoblast Differentiation in MC3T3-E1 Cells

**Figure 2:**
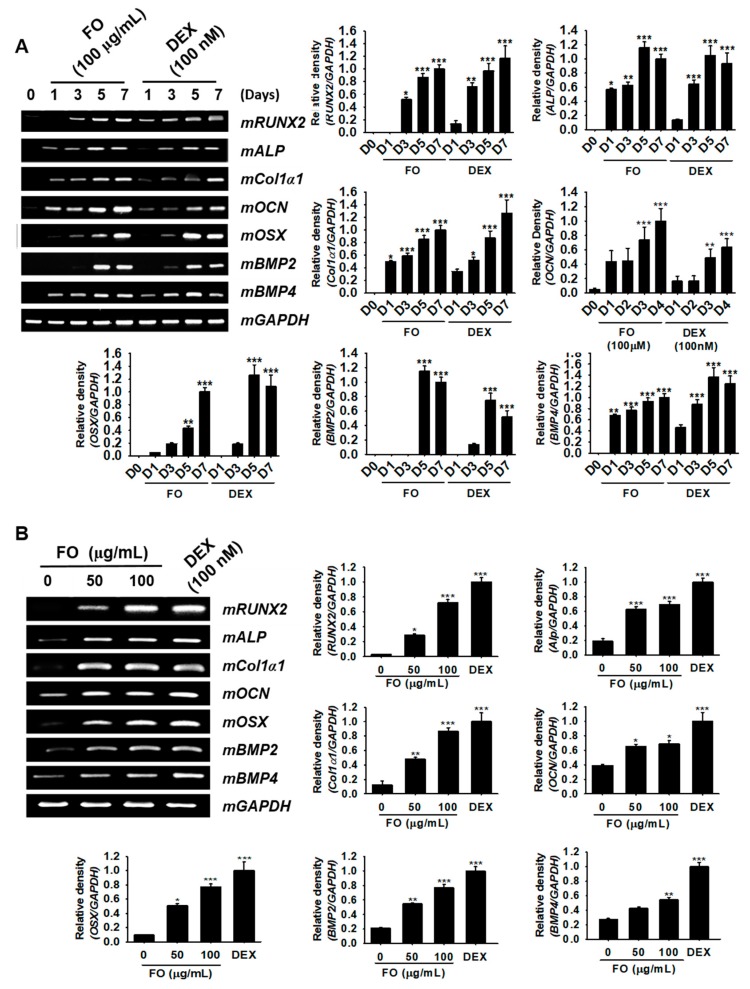
FO upregulates the specific marker gene expression responsible for osteoblast differentiation in MC3T3-E1 cells. (**A**) MC3T3-E1 cells were treated with 100 µg/mL of FO and 100 nM DEX for 7 days. The expression of *mRUNX2*, *mALP*, *mCol1a1*, *mOCN*, *mOSX*, *mBMP2*, and *mBMP4* was measured on day 1 (D1), day 3 (D3), day 5 (D5), and day 7 (D7). (**B**) The cells were also treated with 50 µg/mL and 100 µg/mL of FO or 100 nM DEX for 5 days. The expression of *mRUNX2*, *mALP*, *mCol1a1*, *mOCN*, *mOSX*, *mBMP2*, and *mBMP4*, was observed. *mGAPDH* was used as a house keeping gene. Significant differences among the groups were determined using the one-way ANOVA followed by Bonferroni correction. All data are presented as mean ± SEM (*** *p* < 0.001, ** *p* < 0.01, and * *p* < 0.05 versus untreated group). FO; fermented extract of *C. gigas* and DEX; dexamethasone.

In order to investigate whether FO regulates the expression of osteoblast differentiation-related genes, we treated MC3T3-E1 cells with 100 µg/mL of FO for 7 days. Total mRNA was extracted at day 1, 3, 5, and 7, and then, RT-PCR was performed for osteoblast marker genes such as *mRUNX2*, *mALP*, *mCol1a1*, *mOCN*, *mOSX*, *mBMP2*, and *mBMP4*. We observed that all genes tested in this study exhibited maximal expression at day 7 under both FO- and DEX-treated conditions (Figure 2A). Especially *mALP*, *mCol1a1*, *mOCN*, and *mBMP4* expression increased significantly from day 1 in response to FO treatment, while *mRUNX2*, *mOSX*, and *mBMP2* were positively expressed from day 3. DEX (100 nM), which was used as a positive control, also showed similar expression patterns as those of the FO-treated group, and *mRUNX2* was especially significantly expressed from day 1. Next, we treated the cells with FO (50 µg/mL and 100 µg/mL) for 5 days and extracted the total mRNA to check its concentration-dependent effect. We observed that all marker genes were significantly upregulated after FO treatment in a dose-dependent manner (Figure 2B). Moreover, the effect of FO at 100 µg/mL was higher than that at 50 µg/mL and was comparable to DEX treatment. Altogether, these results revealed that FO significantly upregulated the expression of the marker genes responsible for osteoblast differentiation in MC3T3-E1 cells.

### 3.4. FO Upregulates ALP Expression and Activity in Preosteoblast MC3T3-E1 Cells

**Figure 3:**
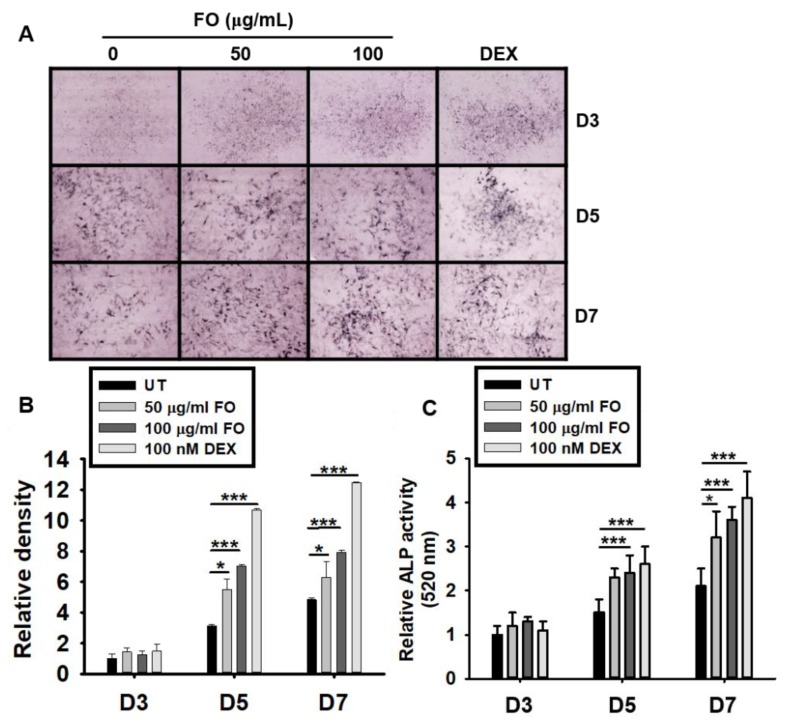
FO upregulates ALP expression and activity in preosteoblast MC3T3-E1 cells. (**A**) MC3T3-E1 cells were treated with FO (50 µg/mL and 100 µg/mL) or DEX (100 nM) for 7 days. Representative images from three biological replicates were collected using the Toup View software after staining the cell for ALP activity (as described in the Materials and Methods section) on day 3 (D3), day 5 (D5), and day 7 (D7). (**B**) The relative ALP staining density was represented using mean integrated pixel density. (**C**) ALP activity was measured at 520 nm using a tartrate-resistant acid phosphatase (TRACP) & alkaline phosphatase (ALP) assay kit. Significant differences among the groups were determined using the one-way ANOVA followed by Bonferroni correction. All data are presented as mean ± SEM (*** *p* < 0.001 and * *p* < 0.05 versus untreated group). FO; fermented extract of *C. gigas* and DEX; dexamethasone.

Although the precise function of ALP is poorly understood, it is believed to play an important role in osteoblast differentiation, which leads to skeletal mineralization [26]. Therefore, we investigated whether FO upregulates ALP expression and activity. As shown in Figure 3A,B, FO and DEX exhibited elevated ALP expression levels from days 5 to 7 when compared to the untreated control group. ALP enzymatic activity also increased by approximately 2 folds at day 5 in response to FO treatment (Figure 3C). The effect of DEX on ALP activity was more prominent than that of FO. These results indicate that FO upregulates ALP activity in MC3T3-E1 cells, and thereby, promotes osteoblast mineralization

### 3.5. FO Increases Osteoblast-Related Protein Expression and Mineralization/Calcification in Osteosarcoma MG-63 Human Osteoblast-Like Cells

**Figure 4:**
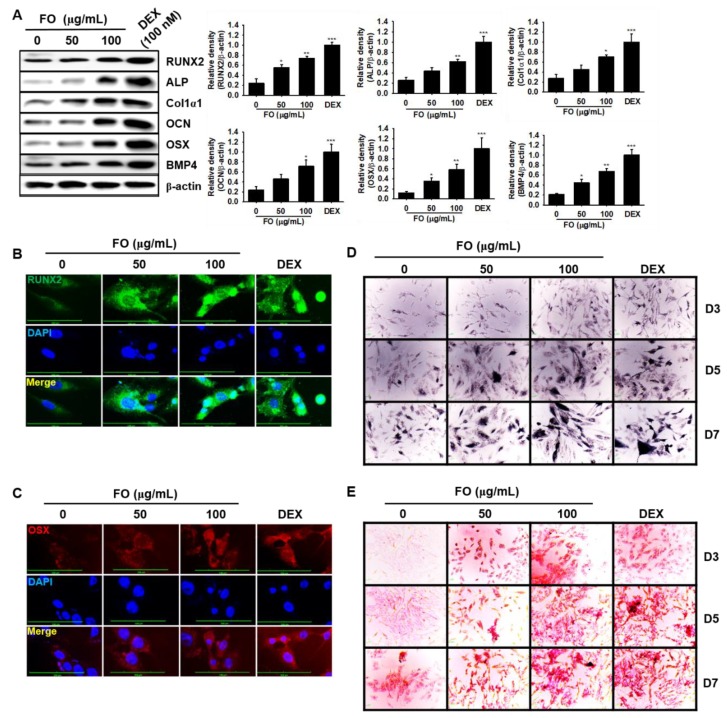
FO activates osteoblast-specific protein expression and mineralization/calcification in osteosarcoma MG-63 human osteoblast-like cells. (**A**) MG-63 cells (3 × 10^3^ cells/mL) were seeded overnight and then treated with FO (50 µg/mL and 100 µg/mL) or DEX (100 nM) for 7 days. Protein was extracted and western blotting analysis was performed using each specific antibody. β-Actin was used as the internal control of protein expression. All protein expression was normalized by the density of β-actin. The nuclear localization of runt-related transcription factor 2 RUNX2 (**B**) and osterix OSX (**C**) was measured by immunostaining. (**D**) ALP activity (mineralization) was measured using a TRACP & ALP assay kit. (**E**) In vitro calcification was detected by alizarin red staining. Significant differences among the groups were determined using the one-way ANOVA followed by Bonferroni correction. All data are presented as mean ± SEM (*** *p* < 0.001, ** *p <* 0.01, and * *p* < 0.05 versus untreated group). FO; fermented extract of *C. gigas* and DEX; dexamethasone. FO; fermented extract of *C. gigas* and DEX; dexamethasone.

Next, to evaluate whether FO increases osteoblastic differentiation of human MG-63 cells, we investigated the expression of osteoblast-specific marker proteins in MG-63 cells in the presence of FO. As shown in Figure 4A, all the tested proteins, such as RUNX2, ALP, Col1α1, OCN OSX, and BMP4, were significantly upregulated in response to 100 μg/mL of FO at day 7 comparable to that of 100 nM DEX. Since RUNX2 and OSX are considered to be major transcription factors in osteoblastogenesis, we stained MG-63 cells with anti-RUNX2 and anti-OSX monoclonal antibodies using Alexa Fluor^®^ to observe their nuclear localization during FO-mediated osteoblastogenesis. As expected, FO promoted the nuclear translocalization of RUNX2 (Figure 4B) and OSX (Figure 4C), which indicates that RUNX2 and OSX play a vital role in FO-mediated osteoblast differentiation. Furthermore, we investigated the involvement of mineralization and calcification during osteoblast differentiation of MG-63 cells. As we expected, FO significantly upregulated ALP activity (mineralization) (Figure 4D) and calcification (Figure 4E) in MG-63 cells by day 7. Taken together, these data indicate that FO also promotes osteoblast differentiation in osteosarcoma MG-63 human osteoblast-like cells.

### 3.6. FO Promotes Vertebrae Formation in Zebrafish Larvae

**Figure 5:**
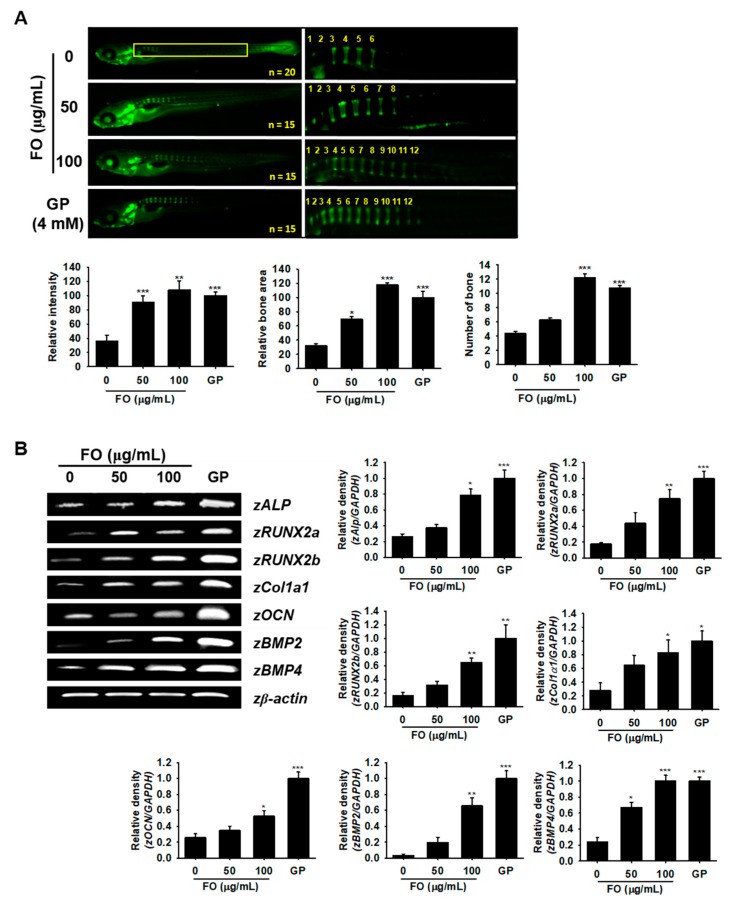
FO promotes vertebrae formation in zebrafish larvae. (**A**) Zebrafish larvae (3 dpf) were treated with FO (50 µg/mL and 100 µg/mL) and GP (4 mM) for 6 days (i.e., until 9 dpf) and then stained with calcein to visualize vertebrae formation. Relative intensity of calcein (bottom, left) and relative bone area (bottom, middle) were calculated using the Image J software and normalized against the GP-treated group. The number of vertebrate in each fish was manually counted. All of them are shown in the yellow box. Total fish length is 3.4 ± 0.4 mm. (**B**) Under the same experimental conditions, total mRNA from the 9 dpf zebrafish larvae was subjected to RT-PCR in order to determine *zALP*, *zRUNX2a*, *zRUNX2b*, *zCol1a1*, *zOCN*, *zBMP2*, and *zBMP4* expression. *zβ-Actin* was used as a house keeping gene. Significant differences among the groups were determined using the one-way ANOVA followed by Bonferroni correction. All data are presented as mean ± SEM (*** *p* < 0.001, ** *p <* 0.01, and * *p* < 0.05 versus untreated group). FO; fermented extract of *C. gigas* and GP; β-glycerophosphate.

Since FO induced osteoblast differentiation at the cellular level, we further assessed this effect in zebrafish larvae. We treated 3 dpf zebrafish larvae with FO for 6 days (i.e., from 3 to 9 dpf) and investigated its effect on vertebrae development. GP, well known to act as a simple phosphate donor and classical serine-threonine phosphatase inhibitor [27], was used as a positive control for these experiments. Vertebrate mineralization in 9 dpf zebrafish larvae was observed via calcein staining, which showed a significantly higher number of FO-induced vertebrae (12.2 ± 0.6 at 100 µg/mL) when compared to the untreated control group (5.4 ± 0.3) accompanied by high calcein staining intensity and total bone area (Figure 5A). FO at 50 µg/mL did not show a significantly higher number of bones (6.3 ± 0.5); however, the calcein fluorescence intensity and relative bone area in the vertebrae significantly increased when compared to the untreated group. Moreover, FO at 100 µg/mL showed a comparable effect with that of the GP-treated group under all the tested parameters, which indicated FO as a possible candidate in strengthening bone formation. For further confirmation, we extracted mRNA from 9 dpf zebrafish larvae and used them as templates for RT-PCR to check the expression of osteoblast differentiation-related marker genes such as *zALP*, *zRUNX2a*, *zRUNX2b*, *zCol1α1*, *zOCN*, *zBMP2*, and *zBMP4*. We found that FO at 100 µg/mL and GP at 4 mM significantly increased the expression of all the tested osteoblast marker genes (Figure 5B), while FO at 50 µg/mL moderately increased the expression of all osteoblast differentiation-related genes when compared to the untreated control. These results indicated that FO induced vertebrae formation in zebrafish via the promotion of bone-related gene expression.

### 3.7. FO Increases Caudal Fin Regeneration in Adult Zebrafish

**Figure 6:**
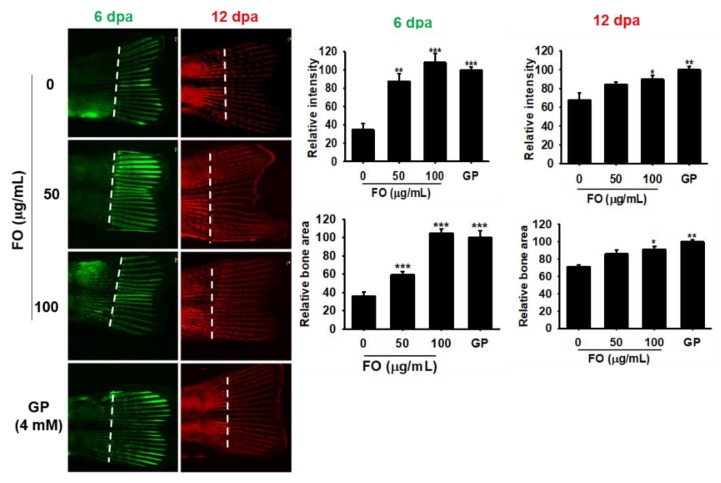
FO promotes caudal fin regeneration in adult zebrafish. Adult zebrafish (*n* = 15) were immersed in FO for 12 days post amputation (dpa). Next, fish caudal fins were subjected to double fluorochrome labeling with calcein and alizarin red at 6 and 12 dpa, respectively. Bone regions labeled with calcein (green) and alizarin red (red) indicate new bone regeneration at 6 and 12 dpa. Relative intensity of each bone vein was calculated using the Image J software. Amputation axis is also indicated (dashed line). Significant differences among the groups were determined using the one-way ANOVA followed by Bonferroni correction. All data are presented as mean ± SEM (*** *p* < 0.001, ** *p* < 0.01, and * *p* < 0.05 versus untreated group). FO; fermented extract of *C. gigas* and GP; β-glycerophosphate.

Unlike mammals, zebrafish can completely and repeatedly regenerate lost appendages such as fins. Fin regeneration occurs via the formation of a progenitor cell population, called the blastema, which contains precursors required by the regenerating tissue [28]. Therefore, we investigated the effect of FO on caudal fin regeneration in adult zebrafish. Using calcein staining, we observed that FO at 50 µg/mL and 100 µg/mL significantly increased the relative intensity and relative bone area by three-fold during caudal fin regeneration when compared to untreated control at 6 dpa; however, the effect was not significant when compared the 4 mM GP treatment (Figure 6, green fluorescence). Both concentrations of FO markedly enhanced the fluorescence intensity (alizarin red staining) in zebrafish tail at 12 dpa (Figure 6, red fluorescence); however, the fold changes of both relative intensity and relative bone area (approximately 1.2 and 1.4 folds at 50 µg/mL and 100 µg/mL of FO, respectively) were smaller than that of day 6. The effect of 4 mM GP (both relative intensity and relative bone area) was almost similar (1.5 folds) to that of 100 µg/mL of FO at day 12, thus suggesting FO to be a potential candidate for bone regeneration during the early stage on damaging.

### 3.8. FO Enhances Osteogenesis via Crosstalk with the Canonical Wnt/β-Catenin Pathway

**Figure 7:**
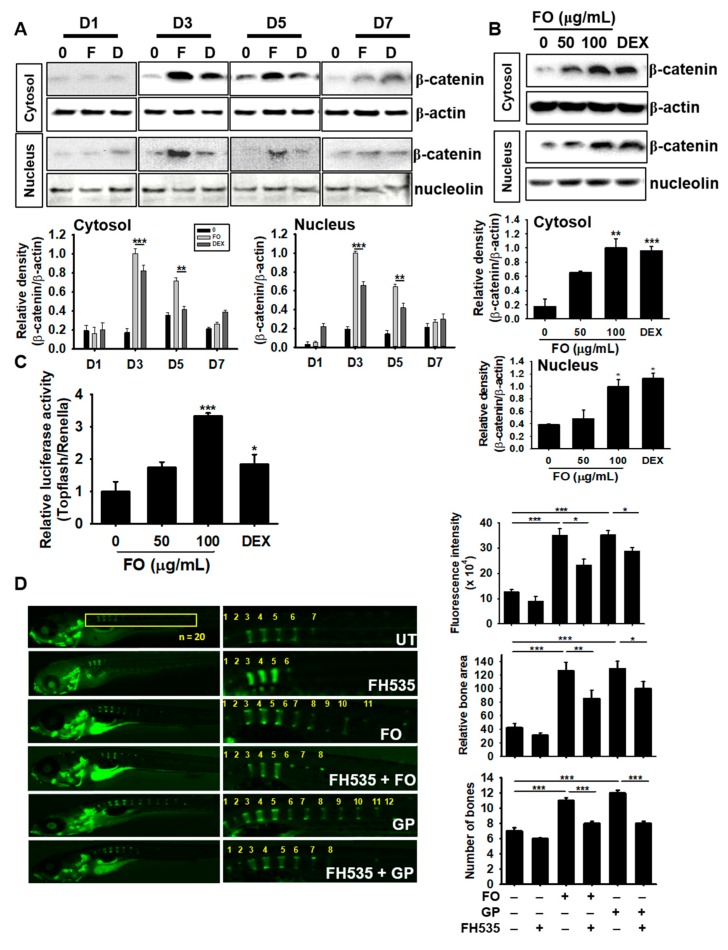
FO enhances osteogenesis via crosstalk with the Wnt/β-catenin pathway. MC3T3-E1 cells were treated with 100 µg/mL of FO and 100 nM DEX for 7 days. (**A**) The cells were harvested at day 1 (D1), day 3 (D3), day 5 (D5), and day 7 (D7). Next, western blot analysis was performed to quantify β-catenin expression. (**B**) The cells were treated with 50 µg/mL and 100 µg/mL of FO or 100 nM DEX for 3 days. Total protein was extracted to quantify β-catenin expression in the cytosol and nucleus. (**C**) To evaluate the effect of β-catenin/TCF signaling, TOPFlash activity was determined. (**D**) Zebrafish larvae (3 dpf; *n* = 20) were pretreated with FH535 for 24 h and then treated with 100 µg/mL of FO or 100 nM DEX. The larvae were stained with calcein to observe vertebrae formation and mineralization 10 dpf. Relative calcein fluorescence intensity and total bone area were quantified using the Image J software and the number of vertebrae was manually counted. Significant differences among the groups were determined using the one-way ANOVA followed by Bonferroni correction. All data are presented as mean ± SEM (*** *p* < 0.001, ** *p* < 0.01, and * *p* < 0.05 versus untreated group). FO; fermented extract of *C. gigas*, DEX; dexamethasone, GP; β-glycerophosphate, dpf; days post fertilization, and UT; untreated group.

The Wnt/β-catenin pathway is not only important for mineralization and development of bone, but is also crucial in modulating skeleton formation [29]. Therefore, we investigated the role of Wnt/β-catenin on FO-induced osteogenesis. We treated MC3T3-E1 cells with FO and DEX for 7 days and examined the expression of β-catenin. FO markedly increased the expression and nuclear translocation of β-catenin at day 3, which gradually decreased but was sustained by day 7, thus indicating that β-catenin may act as an upstream molecule in FO-induced osteoblast differentiation (Figure 7A). FO at 50 µg/mL also moderately increased the expression and nuclear translocation of β-catenin at day 3, but the levels were lower than that of 100 µg/mL of FO or 100 nM DEX treatments (Figure 7B). Next, we transfected MC3T3-E1 cells with TOPFlash luciferase and renilla vectors, and verified Wnt/β-catenin functionality in response to FO. Consistent with western blot analysis, FO was observed to significantly increase β-catenin activity in a dose-dependent manner; however, low FO concentrations (50 µg/mL) only moderately increased this activity (Figure 7C). For further confirmation, we pretreated 3 dpf zebrafish larvae with the Wnt/β-catenin inhibitor, FH535, for 24 h and then treated them with 100 µg/mL of FO and 4 mM GP to 9 dpf. Calcein staining showed that FH535 pretreatment significantly downregulated FO- and GP-mediated vertebrae formation and mineralization in zebrafish larvae by 1.2 and 1.4 folds, respectively (Figure 7D). Altogether, these results suggest that FO-induced osteoblast differentiation and bone formation greatly depends on the canonical β-catenin signaling pathway.

## 4. Discussion

**Figure 8:**
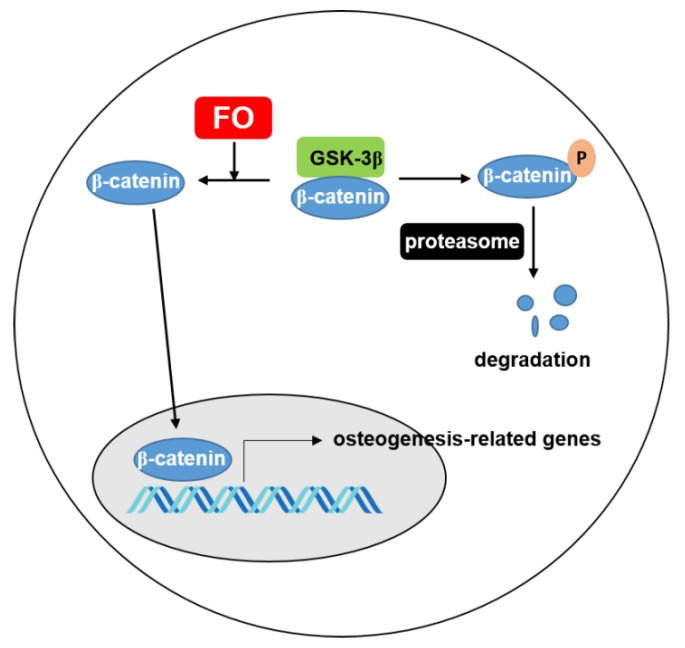
FO increases the expression of β-catenin, which translocates to the nucleus to transactivate osteogenesis-related genes. Under normal condition, β-catenin is phosphorylated by GSK-3β and consequently degraded through proteasome. FO inhibits degradation of β-catenin and increases the movement of β-catenin to nucleus. FO; fermented extract of *C. gigas* and GSK-3β; glycogen synthase kinsase.

Oyster hydrolysate is well known to be a potential resource that exhibits anti-oxidant [30], anti-melanogenic [31], anti-cancerous [32], and anti-viral activities [33]. Many studies have investigated nacre from oyster shells as a bone substitute because it consists of organic and inorganic composite materials that are similar to the basic structure found in mammals [18,19]. Interestingly, the bone substitute from oysters presented potent biocompatibility and stimulated bone-forming cells in an animal model [18]. Additionally, Oliveira et al. [34] identified potential osteogenic proteins from the water-soluble matrix proteins of *C. gigas*. Recently, Chen et al. [20] predicted that novel peptides from *C. gigas* promoted osteogenesis by binding to integrin α5β1. However, none of these studies showed the molecular activity in vitro or in vivo. Previously, our data confirmed that FO inhibited RANKL-mediated ROS generation, which led to the inhibition of osteoclastogenesis [22]. Because FO is rich in protein, FO could promote osteogenesis. However, whether FO directly increases bone formation has not yet been properly understood. In this study, using both in vitro and in vivo approaches, we found that FO potently stimulated preosteoblast MC3T3-E1 cell differentiation of and promoted vertebrae formation and fin regeneration in a zebrafish model by activating the Wnt/β-catenin signaling pathway (Figure 8).

RUNX2 is an inevitable transcription factor of osteoblast differentiation that is produced when mesenchymal stem cells differentiate into immature osteoblasts, thereby leading to the expression of bone formation-related genes such as *ALP*, *Col1a1*, *OCN*, *OSX*, *BMP2*, and *BMP4*. Its expression is found to decrease in mature osteoblasts [35]. Osteoblast differentiation was found to occur normally in *RUNX2*^+^*OSX*^−^ mesenchymal cells, while the same was inhibited in *RUNX2*-deficient embryos, which suggests that RUNX2 is a crucial transcription factor in differentiating mesenchymal cells to osteoblasts [36]. Additionally, OSX is not only positively regulated by activating RUNX2-mediated BMP [37], but it also inhibits osteoblast differentiation at a late stage [38], which indicates that RUNX2 induces osteoblast differentiation from mesenchymal stem cells along with OSX expression at an early stage and that RUNX2-mediated OSX consequently finalizes osteoblast differentiation. Furthermore, *Col1α1* is responsible for the synthesis of type 1 collagen protein, which ensures that the bone and cartilage can resist tensile, shear, and compression forces [39]. Abnormalities in collagen production leads to bone-related diseases such as Paget’s disease and osteoporosis [40]. OCN is the most abundant osteoblast-specific non-collagenous protein involved in bone matrix organization that is, not crucial for bone development [41]. In this study, we found that FO markedly increased osteoblast differentiation marker gene expression, and thereby, promoted the osteoblast differentiation and bone formation. Nevertheless, further research is needed to determine how FO stimulates *RUNX2* at the early stages. Additionally, we found that some osteoblastic genes, such as *BMP4*, *OCN*, *Col1αl*, and *ALP*, showed early expression when compared to *RUNX2*, which indicated that upstream molecules increased some amount of osteogenic gene expression along with RUNX2.

Furthermore, Wnt/β-catenin signaling directly stimulates *RUNX2* and *OSX* expression and osteoblastic genes via the canonical and non-canonical pathways, ultimately leading to bone formation [42,43]. In the absence of Wnt, GSK-3β induces the phosphorylation and degradation of β-catenin. In the presence of Wnt, β-catenin is released from GSK-3β complex with the help of Axin and APC, and consequently, the accumulated free β-catenin translocates to the nucleus and initiates T-cell factor/lymphoid enhancer factor (TCF/LEF)-mediated gene expression [44]. In the current study, FO strongly induced β-catenin expression in the cytosolic compartment, which translocated into the nucleus of MC3T3-E1 cells. Furthermore, FH535, a Wnt/β-catenin inhibitor, decreased vertebrae formation in zebrafish larvae, even though we are unsure how FO stimulated the Wnt/β-catenin signaling pathway. Notably, Brogi et al. [45] newly designed decapeptides that function as agonists of the canonical Wnt/β-catenin signal pathway, which could be potential therapeutic agents in the treatment of bone diseases. Integrin priming using Arg-Gly-Asp (RGD)-containing peptides was also found to promote osteoblast differentiation accompanied with bone mass and microarchitecture improvement by activating the canonical Wnt/β-catenin signaling pathway [46]. Recently, Chen et al. [20] found that RGD-containing peptides from *C. gigas* hydrolysate enhanced ALP activity in preosteoblast MC3T3-E1 cells and predictively bound to integrin α5β1. Based on these studies, we postulated that FO promoted Wnt/β-catenin-mediated osteoblast differentiation and bone formation in preosteoblast cells and zebrafish larvae by binding to Wnt/β-catenin-stimulating receptors such as integrin.

## 5. Conclusions

In conclusion, FO significantly boosts Wnt/β-catenin-mediated osteoblast differentiation and bone formation. Therefore, FO can be considered to be a potential food remedy for bone defects.

## Supplementary Materials

The following are available online at https://www.mdpi.com/2218-273X/9/11/711/s1, Figure S1: UPLC chromatogram for amino acids of FO, Table S1: Proximate composition of FO, Table S2: Amino acid content in FO.
