# Supplementary material for: Fermented Oyster Extract Promotes Osteoblast Differentiation by Activating the Wnt/β-Catenin Signaling Pathway, Leading to Bone Formation
Source: Biomolecules. 2019 Nov 6;9(11):711. doi: 10.3390/biom9110711 (PMC6920898; doi:10.3390/biom9110711)
Supplement: Supplementary file 1 [file biomolecules-09-00711-s001.pdf]

Table S1. Proximate composition of FO

FO	(%)
Protein	60.0 ± 0.7
Carbohydrate	36.0 ± 0.7
Lipid	3.3 ± 0.1
Ash	0.7 ± 0.1

Table S2. Amino acid content in FO

Amino acids	Concentration (%)
Aspartic acid	5.3
Glutamic acid	6.7
Serine	0.9
Histidine	0.9
Glycine	2.9
Threonine	2.6
Arginine	1.9
Alanine	5.0
Taurine	3.0
Tyrosine	1.7
Valine	3.0
Methionine	1.0
Phenylanine	2.0
Isoleucine	2.6
Leucine	3.7
Lysine	29.2
Proline	3.1
GABA*	24.5
Total	100

* GABA; Gamma aminobutyric acid

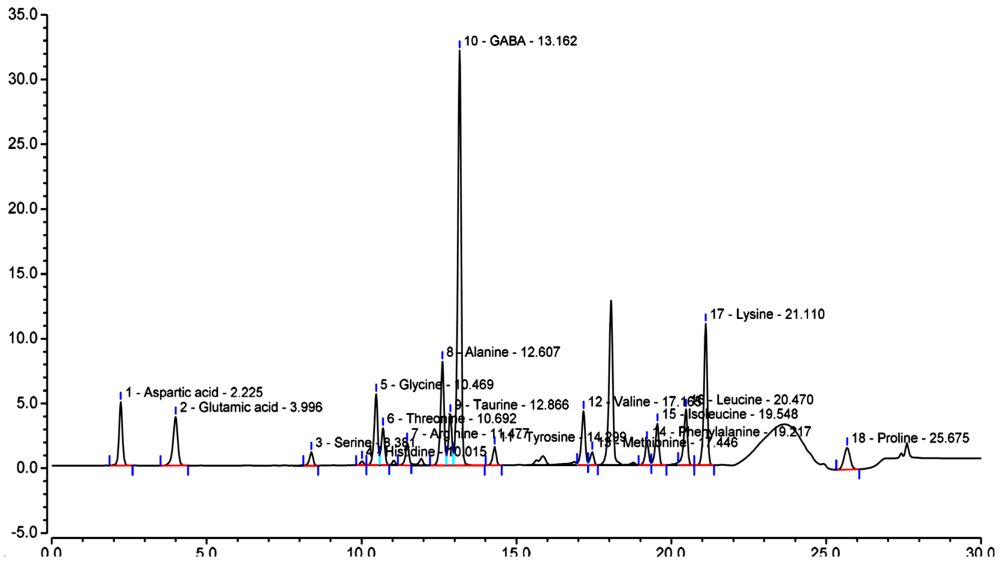

Figure S1. UPLC chromatogram for amino acids of FO.
